# Shiga toxin-producing *escherichia coli* infections in Norway, 1992–2012: characterization of isolates and identification of risk factors for haemolytic uremic syndrome

**DOI:** 10.1186/s12879-015-1017-6

**Published:** 2015-08-11

**Authors:** Lin T. Brandal, Astrid L. Wester, Heidi Lange, Inger Løbersli, Bjørn-Arne Lindstedt, Line Vold, Georg Kapperud

**Affiliations:** Department of Foodborne Infections, The Norwegian Institute of Public Health, Oslo, Norway; Department of Infectious Disease Epidemiology, The Norwegian Institute of Public Health, Oslo, Norway; Gene Technology Section, Akershus University Hospital, Lørenskog, Norway; Division of Infectious Disease Control, The Norwegian Institute of Public Health, Oslo, Norway; Department of Food Safety and Infection Biology, Norwegian University of Life Sciences, Oslo, Norway; Division of Infectious Disease Control, Department of Foodborne Infections, Norwegian Institute of Public Health, P.O. Box 4404, Nydalen, N-0403 Oslo Norway

## Abstract

**Background:**

Shiga toxin-producing *E. coli* (STEC) infection is associated with haemolytic uremic syndrome (HUS). Therefore Norway has implemented strict guidelines for prevention and control of STEC infection. However, only a subgroup of STEC leads to HUS. Thus, identification of determinants differentiating high risk STEC (HUS STEC) from low risk STEC (non-HUS STEC) is needed to enable implementation of graded infectious disease response.

**Methods:**

A national study of 333 STEC infections in Norway, including one STEC from each patient or outbreak over two decades (1992–2012), was conducted. Serotype, virulence profile, and genotype of each STEC were determined by phenotypic or PCR based methods. The association between microbiological properties and demographic and clinical data was assessed by univariable analyses and multiple logistic regression models.

**Results:**

From 1992 through 2012, an increased number of STEC cases including more domestically acquired infections were notified in Norway. O157 was the most frequent serogroup (33.6 %), although a decrease of this serogroup was seen over the last decade. All 25 HUS patients yielded STEC with *stx2*, *eae*, and *ehxA.* In a multiple logistic regression model, age ≤5 years (OR = 16.7) and *stx2a* (OR = 30.1) were independently related to increased risk of HUS. *eae* and hospitalization could not be modelled since all HUS patients showed these traits. The combination of low age (≤5 years) and the presence of *stx2a*, and *eae* gave a positive predictive value (PPV) for HUS of 67.5 % and a negative predictive value (NPV) of 99.0 %. SF O157:[H7] and O145:H?, although associated with HUS in the univariable analyses, were not independent risk factors. *stx1* (OR = 0.1) was the sole factor independently associated with a reduced risk of HUS (NPV: 79.7 %); *stx2c* was not so.

**Conclusions:**

Our results indicate that virulence gene profile and patients’ age are the major determinants of HUS development.

**Electronic supplementary material:**

The online version of this article (doi:10.1186/s12879-015-1017-6) contains supplementary material, which is available to authorized users.

## Background

Shiga toxin-producing *Escherichia coli* (STEC), also called verocytotoxin-producing *E. coli* (VTEC), can lead to mild, self-limiting diarrhoea, haemorrhagic colitis or the life threatening complication haemolytic uremic syndrome (HUS). Children less than five years of age, the elderly, and immunocompromised persons, are most susceptible to STEC infection as well as to severe complications [1]. An association between the Shiga Toxin-encoding gene *stx2*, particularly the subtypes *stx2a*, *stx2c*, and *stx2d*, and development of HUS has been described [2–10]. Several other virulence factors that contribute to the pathogenicity of STEC have been identified, such as *eae* (*E. coli* attaching and effacing) encoding intimin and the plasmid-borne *ehxA* encoding enterohaemolysin [11]. In several parts of the world O157 is the predominating STEC serogroup, and this variant has most frequently been associated with HUS and outbreaks [12–16]. In other countries, however, like continental Europe and Scandinavia, non-O157 serogroups are dominating [3, 17–20]. The involvement of STEC in serious outbreaks combined with a high disease burden per case [21] makes STEC a significant challenge to public health.

In 1995, STEC infection was made mandatory notifiable to the Norwegian Surveillance System for Communicable Diseases (MSIS) (http://www.msis.no), and in 2006 diarrhoea-associated HUS became notifiable. Norway has implemented strict guidelines for prevention and control of STEC infection, in which 3–5 negative stool cultures are required for high-risk groups [22].

A few previous studies have investigated risk factors for development of HUS, however these studies have mainly focused on clinical and demographic parameters among patients infected with either serogroup O157 or O104 [23–25]. Although it is well documented that the presence of *stx2* and *eae* as well as being a child are risk determinants of HUS development, few studies have performed multivariable analyses of both O157 and non-O157 STEC to identify factors independently associated with HUS. Furthermore, knowledge of factors independently associated with reduced risk of HUS is sparse.

The main aim of the present study was to identify microbiologic and patient-related criteria differentiating HUS STEC from non-HUS STEC, in order to obtain information enabling revision of the strict control and prevention measures presently employed in Norway. The second aim was to describe human STEC infections in Norway during two decades (1992–2012), to compare with studies from other countries and contribute to our understanding of this infection in general.

## Methods

### Surveillance of STEC infections in Norway

**Fig 1:**
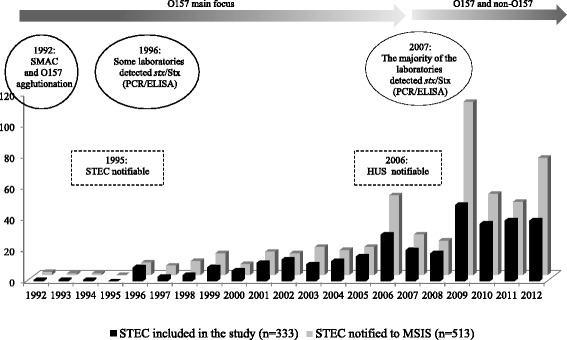
STEC in Norway 1992 to 2012. All STEC infections notified to MSIS during this time period were shown (grey columns, n = 513). Only one STEC isolate per outbreak and per patient was included in the present study (black columns, n = 334 STEC isolates from 333 patients). Before 2006 the main focus of the clinical microbiological laboratories throughout Norway was detection of STEC O157. However, from 2007 the majority of these laboratories implemented techniques for detection of *stx*/Stx and the number of non-O157 cases increased. In 2006, 2009, and 2012, larger STEC outbreaks (>5 cases) were reported (Additional file 1)

Epidemiological and clinical information about STEC infection in Norway from 1992 through 2012 was obtained from MSIS at the National Institute of Public Health (NIPH), which has received mandatory notifications from medical microbiological laboratories and physicians in the country since 1995 (http://www.msis.no/). During this period, 513 STEC infections were notified (Fig. 1) (annual mean, 0.54 cases per 100.000 populations). Cases were recorded as domestic if the patients did not report foreign travel in the incubation period, and as imported if the patients became ill while being abroad or shortly after their return home.

### Characterization of STEC isolates

All isolates were obtained from the National STEC Culture Collection at the Reference Laboratory for Enteropathogenic Bacteria at the NIPH, which receives all presumptive STEC isolates from medical microbiological laboratories throughout Norway.

We selected one isolate per patient and per outbreak, except for one patient from whom two isolates were included since they showed different virulence gene profiles and genotypes. Likewise, only one isolate was selected if isolates with identical genotype were received within the same time period from two patients with different surnames, but living within the same municipality or county, had attended the same child-care facility, or had identical travel history. Nine isolates were from asymptomatic carriers. In total, 334 STEC isolates from 333 patients (64.8 %, 333/513) were included in the study (Fig. 1).

Eighty-three out of the 334 STEC isolates, all from patients living in one of the 19 counties in Norway, have been described previously [26].

### Serotyping

Presumptive STEC isolates submitted to the Reference Laboratory at NIPH were serotyped by slide agglutination using antisera against 43 different O groups (Institut für Immunpräparate und Nährmedien GmbH Berlin (SIFIN), Germany, and Statens Serum Institute (SSI), Denmark) and eight H groups (SSI, Denmark). Non-agglutinating isolates were re-tested by molecular serotyping, in which PCR was run to amplify the *wzx* or *wzy* genes of 14 O groups (O26, O86, O91, O103, O104, O111, O113, O114, O117, O121, O128, O145, O146, and O157) and the *fliC* gene of 10 H groups (H2, H4, H7, H8, H10, H11, H19, H21, H25, and H28) (Lindstedt *et al.,* unpublished).

### Sorbitol-fermenting (SF) *E. coli* O157

All STEC isolates belonging to serogroup O157 were analysed with a multiplex PCR (M-PCR) specific for SF *E. coli* O157 [27] in order to distinguish SF O157 STEC from the classical non-sorbitol fermenting (NSF) O157 STEC.

### Virulence genes characterization

From 1992 to 2001, production of Stx1 and Stx2 was ascertained using the GM1 ganglioside enzyme-linked immunosorbent assay (GM1-ELISA) with some minor modifications [28]. In 2001 the ELISA was replaced by a M-PCR detecting *stx1* and *stx2* [29]. The same year a PCR detecting *eae* was also included [30, 31]. Since July 2005 an octaplex-PCR [32], later expanded to an endecaplex-PCR, was used for routine screening of all enteropathogenic *E. coli*. This encompassed primers for *stx1*, *stx2*, *eae*, *ehxA*, *bfpB* and *rrs* [33], as well as primers for *ipaH* [34], *LTI* (F-primer; GTT TTA TTT ACG GCG TTA CTA TCC and R-primer; ATT GGG GGT TTT ATT ATT CC), *STIa* [35], *STIb* [35], and *aggR* [34]. All STEC isolates confirmed before July 2005 were re-tested for *eae* and *ehxA* using the PCR primers described by Brandal *et al.* [33].

### *stx* subtyping

Subtypes of *stx1* were identified by PCR as described by Scheutz *et al.* [8]. Subtyping of *stx2* was performed with one of the two following methods. The first method used PCR restriction fragment length polymorphism (RFLP) followed by electrophoresis (modification of Russmann *et al.* [36] and Jelacic *et al.* [37]), and sequencing [7], in which all *stx2d* positive isolates were verified by a *stx2d* specific PCR [38]. The second method determined *stx2* subtypes by PCR as described by Scheutz *et al.* [8].

### Genotyping

The O157 isolates were genotyped by an O157 specific multi-locus variable-number tandem repeat analysis (MLVA) [39]. All non-O157 STEC isolates were genotyped using a seven loci generic MLVA [40] or an updated 10 loci generic MLVA [41].

### Statistical methods

Statistical analyses were performed using the computer program SPSS® release 20.0.0 (IBM SPSS Software, International Business Machines Corp., Armonk New York). Univariable analyses were performed with the procedures for cross tables (dichotomous variables) or comparison of means (continuous variables) as appropriate. Binary logistic regression was implemented for multivariable analyses. The results are reported as odds ratios with 95 % confidence intervals and two-tailed *p* values. A *p* value of ≤ 0.05 was considered statistically significant. Positive and negative predictive values were calculated as described by Altman & Bland [42].

### Ethical considerations

At the NIPH, all STEC strains are routinely collected for disease surveillance and outbreak detection. The current study is based on descriptive analysis of bacterial isolates from the strain collection and the microbiological characteristics so obtained could only be combined with the sex, age, clinical outcome, hospitalization, travel history, and seasonality for the patients from which the strains were isolated. Ethical approval was therefore not required. Also, the Norwegian Communicable Disease Control Act and its companying regulations (https://lovdata.no/dokument/NL/lov/1994-08-05-55?q=Smittevernloven) obliges the NIPH to perform national surveillance of communicable diseases, including STEC infection. For these reasons, consent was not obtained from the patients to analyze the bacterial samples for this research project.

## Results

### STEC infections in Norway

From 1992 to 2006, 0–20 STEC cases were notified each year, whereas from 2006 the number of notified cases increased, ranging from 22 to 111 annually (mean 54.9) (Fig. 1). Of the 513 patients recorded by surveillance, 57 developed HUS (11.1 %), and isolates from 36 of them were submitted to the National Reference Laboratory at NIPH. The major outbreaks reported in Norway during 1992 through 2012 are presented in Additional file 1.

### Demographics and clinical presentation

The mean age of the 333 patients selected for the study was 24.6 years and 39.0 % (130/333) were ≤ 5 years old. Diarrhoea was the most frequent clinical manifestation, and 35.7 % (119/333) reported bloody diarrhoea (Table 1). Twenty-five of the patients (7.5 %) developed HUS (Additional file 2). Furthermore, 49.8 % had reportedly acquired their infection in Norway (Table 1), but a higher proportion of domestically acquired STEC infections was observed from 2006 compared to previous years (127/190, 66.8 % (from 2006) versus 39/83, 46.7 % (before 2006), p < 0.005).

**Table 1:** Association between patient-related factors in O157 versus non-O157 and HUS versus non-HUS STEC infections, Norway 1992–2012

Characteristics		No. (%) of patients						
		All (333)	O157 (112, 33.6 %)	Non-O157 (221, 66.4 %)	p-value^a^	HUS (25, 7.5 %)	Non-HUS (308, 92.5 %)	p-value
Sex	Male	146 (43.8 %)	47	99		9	137	
	Female	187 (56.2 %)	65	122		16	171	
Age group (yr)	≤5	130 (39.0 %)	25	105	<0.005	22	108	<0.005
	6-18	41 (12.3 %)	16	25		2	39	
	19-41	76 (22.8 %)	30	46		0	76	
	42-64	51 (15.4 %)	23	28		0	51	
	≥65	35 (10.5 %)	18	17		1	34	
Age mean		24.6	33.9	20.33	<0.005	4.72	26.2	<0.005
HUS	Yes	25 (7.5 %)	9	16		25	0	
	No	308 (92.5 %)	103	205		0	308	
Clinical outcome	Bloody diarrhea	119 (35.7 %)	40	79		13	106	<0.005
	Diarrhea	148 (44.4 %)	57	91		1	147	<0.005
	Asymptomatic	9 (2.7 %)	0	9		0	9	
	Unknown	57 (17.1 %)	15	42		11	46	
Hospitalized	Yes	141 (42.2 %)	64	77		24	117	
	No	155 (46.4 %)	39	116	<0.005	0	155	<0.005
	Unknown	37 (11.1 %)	9	28		1	36	
Travel history	Domestically	166 (49.8 %)	40	126		16	150	
	Imported	107 (32.1 %)	57	50	<0.005	3	104	
	Unknown	60 (18.0 %)	15	45		6	54	
Seasonality^b^	Summer	118 (35.4 %)	50	68		6	112	
	Autumn	74 (22.2 %)	20	54		9	65	
	Winter	75 (22.5 %)	24	51		7	68	
	Spring	66 (19.8 %)	18	48		3	63	

^a^Only p-values ≤ 0.05 were shown

^b^Summer; June-August, Autumn; September-November, Winter; December-February, Spring; March-May

### Characterization of selected STEC

#### Serogroups

Twenty-four different O groups were identified among 292 of the 334 STEC isolates examined from 333 patients. The remaining 42 isolates were non-typable with the methods employed (one of these was rough). The majority of the isolates (69.5 %, 232/334) were motile, and nine H groups were discerned. Thus, a total of 58 different O and H combinations were identified.

O157 was the most frequent serogroup detected (112/333, 33.6 %) (Table 1). The percentage of serogroup O157 significantly decreased from 49.6 % (65/131) during 1992–2006, to 23.3 % (47/202) in 2007–2012 (p < 0.005). Compared to non-O157, O157 infection was significantly associated with older age, foreign travel prior to disease onset, and a higher rate of hospitalization. No statistical significant differences between O157 and non-O157 infected persons were detected regarding clinical outcome (Table 1).

Serogroup O157 included 103 isolates that were NSF and nine that were SF. NSF O157 infections were more likely to occur during summer and spring, whereas infections with SF O157 were associated with colder months of the year. None of the patients with SF O157 infection reported foreign travel prior to onset of disease (Additional file 3). In a multivariable model, both foreign travel and seasonality (summer) were independently related to NSF O157 infection (OR = 4.4, CI = 2.5-7.5 and OR = 1.9, CI = 1.1-3.3, respectively).

Non-O157 STEC infection was detected in 66.4 % of the patients (221/333) (Table 1). The most common serogroups were O103 (15.0 %), O26 (10.2 %), O145 (7.2 %), O91 (3.9 %), O117 (3.3 %), O121 (2.1 %), O113 (1.8 %), O146 (1.8 %), and O111 (1.2 %). Infection with STEC O103, O26, or O121 was associated with younger age. Additionally, patients infected with O103 or O145 were less likely to report foreign travel prior to infection (Additional file 3).

#### Virulence genes

Of the 334 STEC from 333 patients, 127 (38.1 %) carried *stx1* only, 118 (35.3 %) harboured *stx2* only, and 89 (26.6 %) exhibited both *stx1* and *stx2*. Thus, isolates from 215 patients (64.6 %) were positive for *stx1* and 207 (62.2 %) carried *stx2* (Table 2). None of the *stx1* positive isolates harboured more than one *stx1* subtype. *stx1a* was the most frequently detected subtype. Of the patients with *stx2* positive STEC, 100 (48.3 %) contained *stx2c* and 85 (41.1 %) carried *stx2a* . Nearly three-fourths of the patients had an *eae* positive STEC and the majority of the cases harboured STEC with *ehxA* (Table 2).

**Table 2:** Association between virulence genes, serogroups, HUS, and hospitalization among 333 cases of STEC infection, Norway 1992–2012

Virulence genes		No. (%) of patients								
		All (333)	O157 (112, 33.6 %)	Non-O157 (221, 66.4 %)	p-value^a^	HUS (25, 7.5 %)	Non-HUS (308, 92.5 %)	p-value	Hospitalized^b^(141, 42.3 %)	p-value
*stx1*		215 (64.7 %)	66	150		1^c^	214	<0.005	83	0.01
	*stx1a*	189	66	123		1	188	<0.005	75	
	*stx1c*	23	0	23	<0.005	0	23		7	
	*stx1d*	3	0	3		0	3		1	
*stx2*		207^d^ (62.2 %)	109	98	<0.005	25^e^	182	<0.005	106	<0.005
	*stx2a*	85	32	53		24	61	<0.005	51	<0.005
	*stx2b*	32	0	32	<0.005	0	32		6	0.05
	*stx2c*	100	92	8	<0.005	2	98	0.01	57	<0.005
	*stx2d*	9	1	8		0	9		4	
	*stx2g*	2	0	2		0	2		ND^f^	
*eae*		246 (73.9 %)	112	134	<0.005	25	220	<0.005	117	0.02
*ehxA*		283 (85.0 %)	109	174	<0.005	25	258		131	<0.005

^a^Only p-values ≤ 0.05 were shown

^b^No information on hospitalization was available for 37 patients. These patients carried STEC with the following *stx* genes; *stx1a* (n = 17), *stx1c* (n = 1), *stx1d* (n = 2), *stx2a* (n = 6), *stx2b* (n = 9), *stx2c* (n = 10), and *stx2g* (n = 2) (n = number of patients) (some patients carried STEC with more than one *stx* subtype)

^c^From one HUS patient two different non-O157 STEC isolates were included. One with *stx1a* and another with *stx1a* + *stx2a* and this patient was included both in the *stx1* and *stx1a* groups as well as in the *stx2* and *stx2a* groups

^d^Including 21 patients with STEC harbouring more than one *stx2* subtype; *stx2a* + *stx2c* (n = 19), *stx2a* + *stx2d* (n = 1), and *stx2c* + *stx2d* (n = 1)

^e^One HUS patient carried a STEC with both *stx2a* + *stx2c* and this patient was included within the *stx2a* group as well as in the *stx2c* group

^f^ND: not determined

Patients with O157 and non-O157 STEC did not differ with regard to presence of *stx1* (Table 2). However, when *stx1* was stratified according to subtypes, *stx1c* was more frequently detected among patients with non-O157 STEC, and neither *stx1c* nor *stx1d* was found in any of the patients with O157 isolates (Table 2, Additional file 4).

Nearly all patients with O157 isolates carried *stx2*, while less than half of their non-O157 counterparts had this gene. Among the *stx2* subtypes, *stx2c* and *stx2a* + *stx2c* were both more common in O157 (p < 0.005 for each), whereas *stx2b* and *stx2g* were only detected in the non-O157 group (Table 2, Additional file 4).

Both *eae* and *ehxA* were more frequently detected in O157 compared to non-O157 (Table 2).

Within O157, NSF O157 were associated with *stx2*, *eae* and *ehxA*, whereas SF O157 were less likely to harbour *stx1* (Additional file 3).

Among non-O157 isolates, O103 was more likely to carry *stx1* than the other serogroups combined, while in O145 or O121 *stx1* was infrequent. All O91, O113, and O146 isolates were *eae* negative, and only two of the O117 isolates carried this gene (Additional file 3).

Patients with *stx1* positive STEC showed a reduced risk of hospitalization, whereas the contrary was seen for patients with *stx2*, *eae*, and *ehxA* (Table 2).

#### Discrimination between HUS and non-HUS STEC

All 25 HUS patients, except three, were ≤ 5 years. Compared to non-HUS cases, patients with HUS were more often hospitalized and showed bloody stools (Table 1).

Seven serogroups were found among STEC from HUS patients: O157 (including both NSF O157 and SF O157 isolates), O145, O26, O103, O121, O111, and O86 (Additional file 2), however only serogroups SF O157 and O145 were significantly associated with HUS (Table 3 and Additional file 3). Within serogroup O145, four of the six patients with STEC O145:H? presented with HUS (p < 0.005, PPV:66.7 %, NPV:93.6 %), whereas only one of eighteen patients with O145:H28 showed this complication. No significant association between HUS and serogroup O103 was seen, but all three patients with STEC O103:H25 developed HUS.

**Table 3:** Risk factors for HUS among 333 STEC patients, Norway, 1992–2012

Determinant^a^	No. of patients		Predictive values^b^		Univariable analyses^c^	Multivariable analyses^c, d^		
	All (333)	HUS (25)	PPV	NPV	OR (95 % CI)	Model 1 (risk factors)	Model 2 (preventive factors)	Model 3 (all factors)
						OR (95 % CI)	OR (95 % CI)	OR (95 % CI)
Age ≤ 5 yr	130	22	16.9 %	98.5 %	13.6 (4.0-46.4)	12.2 (3.2-46.7)		16.7 (4.24-65.7)
Bloody diarrhea	119	13	10.9 %	99.4 %	19.5 (2.5-151.3	ND^e^		
Diarrhea	148	1	0.7 %	78.1 %	0.1 (0.008-0.5)		ND	
Hospitalized^f^	141	24	17.0 %	100.0 %	ND			
SF O157	9	5	55.6 %	93.8 %	19.0 (4.7-76.3)	NS^g^		
O145	24	5	20.8 %	93.5 %	3.8 (1.3-11.2)			
O145:H?	6	4	66.7 %	93.6 %	29.1 (5.0-168.4)	NS		
*stx1*	215	1^h^	0.5 %	79.7 %	0.02 (0.002-0.1)		0.02 (0.002-0.1)	0.1 (0.01-0.8)
*stx1a*	189	1^h^	0.5 %	83.3 %	0.03 (0.004-0.2)			
*stx2*	207	25	12.1 %	100.0 %	ND			
*stx2a*	85	24	28.2 %	99.6 %	97.2 (12.9-732.5)	92.7 (10.7-803.5)		30.1 (3.3-271.9)
*stx2c*	100	2	2.0 %	90.1 %	0.2 (0.04-0.8		0.2 (0.03-0.7)	0.6 (0.1-3.3)
*eae*	245	25	10.2 %	100.0 %	ND			

^a^All determinants associated with HUS (p ≤ 0.05) were included

^b^PPV; positive predictive value, NPV; negative predictive value

^c^OR; odds ratio, CI; 95 % confidence interval

^d^Model 1; factors related to increased risk of developing HUS, Model 2; factors related to reduced risk of HUS, and Model 3; comprising both factors related to increased and decreased risk of HUS

^e^ND; not determined since all HUS cases were hospitalized and all HUS isolates carried *stx2* and *eae*

^f^Information on hospitalization was not available for 37 patients, including one HUS patient

^g^NS; not statistically significant

^h^One HUS patients had two STEC isolates; one with *stx1a* + *stx2a* and another with *stx1a* only

All HUS patients carried STEC with *stx2*, *eae*, and *ehxA*, however only *stx2* and *eae* were significantly associated with HUS, while *ehxA* was not (Table 2 and 3). *stx2a* was present in 24/25 HUS STEC (including one with *stx2a* + *stx2c*), whereas the last case carried *stx2c* only. Both *stx1* and *stx2c* were negatively associated with HUS (Table 2 and 3). The combination of age ≤ 5 years and STEC possessing *stx2a* and *eae* showed strong association to HUS development with PPV of 64.7 % and NPV of 99.0 %. Additionally, this combination gave the highest sensitivity (88.0 %) and specificity (96.1 %) of all determinants investigated (data not shown).

Three multivariable models were fitted, with and without including potentially protective factors. In the first model, two factors were found to be independently related to increased risk of developing HUS: *stx2a* (OR = 92.7, CI = 10.7-803.5) and age ≤ 5 years (OR = 12.2, CI = 3.2-46.7). In this model, the following factors were not independently associated with HUS: SF O157 and O145:H?, although they were significant in the univariable analysis (Table 3). No first-order interactions were detected in the model. All HUS patients with bloody diarrhoea carried STEC with *stx2a,* and bloody diarrhoea was therefore not included in the model containing *stx2a*. Furthermore, *eae*, *stx2*, and hospitalization could not be modelled since all HUS patients showed this trait (for one HUS patient no information on hospitalization was available).

In a separate model assessing protective factors only, both *stx1* (OR = 0.02, CI = 0.002-0.1) and *stx2c* (OR = 0.2, CI = 0.03-0.7) were independently associated with reduced risk of HUS (Table 3). Non-bloody diarrhoea was not included in the model as only one HUS patient showed this symptom. In the third model comprising *stx2a*, age, *stx1* and *stx2c*, *stx1* was still related to reduced risk of HUS development (OR = 0.1, CI = 0.01-0.8), whereas *stx2c* was not (OR = 0.6, CI = 0.1-3.3) (Table 3).

## Discussion

Low age (≤5 years), and the presence of STEC with *stx2a* and *eae* were identified as risk factors for HUS development. An association between HUS and these parameters has been seen in several studies [2–10, 16–18, 43], however, only a few have explored this by multivariable analysis [2, 7, 44]. The high NPV (99.0 %) obtained for this combination of determinants indicates that the likelihood of developing HUS is very low when all these factors are negative. However, not all patients with these three risk factors developed HUS (PPV of 64.7 %), emphasizing that other strain characteristics or host specific factors, like the patient’s immunocompetence, also are important to consider when assessing a patients’ risk for developing HUS. Bloody diarrhoea has previously been identified as a risk factor for HUS [2], and a similar association was achieved in our study, although not proven as an independent risk factor. Interestingly, when only including protective factors in a multivariable model, *stx2c* was independently associated with reduced risk of HUS, but not when both protective and risk factors were included in the same model. The role of *stx2c* in HUS pathogenesis has been debated and it has been speculated that *stx2c* merely assists *stx2a* during development of this severe complication [7]. However, our results indicated that *stx2c* neither was sufficient nor necessary for HUS development. In one of the two HUS patients with *stx2c* positive STEC, *stx2c* and *stx2a* were both present, whereas in the second case *stx2c* was the sole *stx* gene detected. It is possible, though, that this isolate had lost the *stx2a* encoding bacteriophage, a phenomenon previously described in isolates from HUS patients [45, 46]. *stx1* was independently related to reduced risk of HUS in two multivariable models. This has to our knowledge never been shown before, although such an association has been suggested [3, 16, 26, 47–50]. None of the HUS patients carried STEC with *stx1* as the sole *stx* gene present. The single HUS patient with *stx1* yielded two O111:[H8] isolates, one with *stx1a* + *stx2a* and the other with *stx1a* only, indicating that *stx2a* was the *stx* gene responsible for HUS development. Recently, it was demonstrated that STEC O111:H8 strains frequently lose their *stx2* encoding bacteriophage during *in vitro* growth, suggesting that this loss may occur *in vivo* as well [3, 51]. Moreover, *stx1* showed a low PPV for HUS, a finding which further emphasises that *stx1* was not a key factor for HUS development.

In contrast to some authors [3, 15, 16, 52], but in concordance with others [2, 7], we did not find any significant difference between STEC O157 and non-O157 regarding HUS. Interestingly, of the O157 STEC isolated from HUS patients, SF O157 was the dominating variant, despite the fact that NSF O157 was the most frequent STEC detected in Norway. A high frequency of SF O157 in HUS cases has also been reported from other European countries [9, 53] and it has been suggested that patients with STEC SF O157 more often develop HUS compared to patients with NSF O157 [54, 55]. Furthermore, SF O157 and O145 (particularly O145:H?) were the only serogroups associated with HUS in our univariable analyses, although they were not significant in the multivariable models. All STEC O145:H? and SF O157 cases were domestically acquired, indicating a reservoir of these bacteria in Norway. Both serogroups have previously been responsible for HUS outbreaks in our country [56].

Our results confirm that the severity of STEC illness depends strongly on the virulence gene profile of the infecting STEC as well as the patients’ age, unlike serogroup affiliation [2, 57, 58]. Nevertheless, exceptions exist and therefore clinical findings and the epidemiological situation of each STEC case have to be considered before proper control and prevention measures can be implemented.

In Norway infections with non-O157 STEC were more common than infections with O157 isolates, in accordance with findings from several other countries [3, 17–19, 59–61]. Expectedly, the proportion of STEC O157 declined compared to non-O157 from approximately 2007, when improved methods for detecting *stx*/Stx were implemented in the majority of clinical microbiological laboratories in Norway [3, 16, 50, 61, 62]. In contrast to reports from other countries, more than half of the STEC O157 infections in Norway were imported and no seasonal differences between O157 and non-O157 infections were seen [16, 63, 64]. Since ruminants are the main reservoir of STEC O157 [65], the low prevalence of STEC O157 among ruminants in Norway might explain these findings [66–69]. Non-O157 infections were more frequently seen in children (≤5 years) and were more often domestically acquired than O157 infections. Contact with ruminants has previously been identified as the strongest risk factor for non-O157 infection in young children [70] and the following data indicate that this might be the case also in Norway: A national survey of Norwegian sheep flocks [69] showed that as many as 17.3 % (85/491) of the flocks carried non-O157 *E. coli* considered to be human pathogens (unpublished data). Also, non-O157 STEC outbreaks associated with sheep contact or eating mutton have been reported in Norway [71, 72].

There are some limitations to our study. Firstly, we did not examine a consecutive series of STEC isolates from the National STEC Culture Collection, but selected one STEC per patient and per outbreak. Therefore a correct incidence of STEC isolates was not achieved. However, the main aim of our study was to define factors discriminating HUS-STEC from non-HUS STEC. Inclusion of all STEC isolates would have given a biased contribution of the different parameters due to overrepresentation of isolates involved in outbreaks. Secondly, it is likely that non-O157 STEC were underestimated before 2007 since the sensitivity of diagnostic methods were suboptimal at that time. Although the laboratory methods have improved, non-O157 STEC isolation is still a diagnostic challenge due to lack of a selective growth media with sufficient sensitivity. Thirdly, the number of HUS cases included in the study was too low to identify other than the strongest risk factors. Finally, the available clinical information did not permit detailed analysis of patient-related factors such as underlying illnesses, antibiotic treatment, and co-infections, all of which have been considered as putative risk factors for HUS.

## Conclusions

Our results showed that the characteristics of the Norwegian STEC isolates were in concordance with data from other countries. However, some country specific characteristics were unravelled. Multivariable regression analyses identified low age (≤5 years) and the presence of *stx2a* as independent risk factors for HUS development. Additionally, all HUS STEC carried *eae*. On the other hand, *stx1* was independently associated with reduced risk of HUS. Hence, the virulence profile and the patients’ age - but not particular serogroups - were the essential determinants discriminating HUS STEC from non-HUS STEC. The results achieved from the current study will contribute, together with previous published knowledge, to revision of the strict national guidelines for prevention and control of STEC infections currently applied in Norway. Nevertheless, it should be emphasized that in addition to the risk factors identified, the clinical presentation of each patient and the epidemiological context also should be taken into account before advice of control and prevention can be given.

## Additional files

Additional file 1:
**Reported outbreaks of human STEC infections, Norway 1992–2012.** Characteristics of both local and nationwide STEC outbreaks detected in Norway from 1992–2012. Additional file 2:
**STEC isolates from HUS patients included in the present study, Norway 1992–2012.** Microbiological characteristics of STEC isolates (n = 26) from haemolytic uremic syndrome (HUS) patients and characteristics of patients with HUS (n = 25) in Norway from 1992–2012. Additional file 3:
**Characteristics associated with the most common STEC serogroups, Norway 1992–2012.** Demographic, clinical, and virulence characteristics among STEC harbouring the most common serogroups, Norway from 1992–2012. Additional file 4
**Distribution of** ***stx*** **genotypes in STEC O157 compared to non-O157 STEC, Norway 1992–2012.** Distribution and combination of *stx1* and *stx2* subtypes in STEC O157 compared to non-O157 STEC, Norway from 1992–2012.

## Abbreviations

CI: Confidence interval
*eae*: *E. coli* attaching and effacing
*ehxA*: Enterohaemolysin
HUS: Haemolytic uremic syndrome
NIPH: Norwegian Institute of Public Health
NPV: Negative predictive value
NSF: Non-sorbitol fermenting
MLVA: Multiple-locus variable-number of tandem repeat analysis
MSIS: The National Surveillance System for Communicable Diseases
OR: Odds ratio
PPV: Positive predictive value
SF: Sorbitol-fermenting
STEC: Shiga toxin-producing *E. coli*
*Stx*: Shiga toxin
VTEC: Verocytotoxigenic *E. coli*
