# Supplementary material for: Shiga toxin-producing escherichia coli infections in Norway, 1992–2012: characterization of isolates and identification of risk factors for haemolytic uremic syndrome
Source: BMC Infect Dis. 2015 Aug 11;15:324. doi: 10.1186/s12879-015-1017-6 (PMC4531490; doi:10.1186/s12879-015-1017-6)
Supplement: Additional file 1: — Reported outbreaks of human STEC infections, Norway 1992–2012. Characteristics of both local and nationwide STEC outbreaks detected in Norway from 1992–2012. [file 12879_2015_1017_MOESM1_ESM.docx]

**Additional file 1** Reported outbreaks of human STEC infections, Norway 1992-2012^1^.

| **Year** | **Serotype** | **Virulence genes** | **Local/Nationwide**^2^ | **No. of ill persons** | **Suspected vehicle** | **No. of persons with HUS** | **Reference** |
| --- | --- | --- | --- | --- | --- | --- | --- |
| 1999 | O157:H7 | *stx2a*, *eae*, *ehxA* | Nationwide | 4 | Lettuce | 1 | - |
| 2003 | O157:H7 | *stx1a*, *stx2c*, *eae*, *ehxA* | Local | 5 | - | 0 | - |
| 2006 | O103:H25^3^ | *stx2a*, *eae*, *ehxA* | Nationwide | 17 | Cured sausage | 10 | [[1](#_ENREF_1)] |
| 2009 | O145:H28^4^ | *stx1a*, *eae*, *ehxA* | Local | 16 | - | 0 | [[2](#_ENREF_2)] |
| 2009 | SF O157:[H7]^5^ | *stx2a*, *eae*, *ehxA* | Nationwide | 13 | - | 9 | [[3](#_ENREF_3), [4](#_ENREF_4)] |
| 2009 | O121:H19 | *stx2a*, *eae*, *ehxA* | Local | 3 | - | 1 | - |
| 2009 | O145:H? | *stx2a*, *eae*, *ehxA* | Nationwide | 3 | - | 3 | - |
| 2009 | O103:H2 | *stx1a*, *eae*, *ehxA* | Nationwide | 7 | - | 0 | - |
| 2010 | SF O157:[H7]^6^ | *stx2a*, *eae*, *ehxA* | Nationwide | 3 | - | 3 | - |
| 2012 | O103:H2^7^ | *stx1a*, *eae*, *ehxA* | Local | 7 | - | 0 | [[5](#_ENREF_5)] |
| 2012 | O?:H? | *stx1d* | Local | 3 | - | 0 | - |

^1^An outbreak is defined as three or more persons infected with STEC showing identical MLVA-profile within the same time period. Family outbreaks are not included in this table.

^2^Nationwide; an outbreak is defined as nationwide if persons from three or more counties in Norway are involved.

^3^The outbreak strain was recovered from 11 of 17 persons involved in the outbreak. Only two of the 11 isolates harboured *stx2a*.

^4^In one of the outbreak isolates, *ehxA* was not detected*.*

^5^The outbreak strain was a sorbitol fermenting (SF) *E. coli* O157:[H7]. Three of the isolates lacked *stx2a*.

^6^This SF O157:[H7] strain was identical to the outbreak strain in 2009.

^7^The MLVA-profile of the outbreak strain differed in two loci from the O103:H2 outbreak strain in 2009. One of the isolates lacked *ehxA.*

**References**

1. Schimmer B, Nygard K, Eriksen HM, Lassen J, Lindstedt BA, Brandal LT, Kapperud G, Aavitsland P: **Outbreak of haemolytic uraemic syndrome in Norway caused by stx2-positive Escherichia coli O103:H25 traced to cured mutton sausages.** *BMC Infect Dis* 2008, **8:**41.

2. Wahl E, Vold L, Lindstedt BA, Bruheim T, Afset JE: **Investigation of an Escherichia coli O145 outbreak in a child day-care centre--extensive sampling and characterization of eae- and stx1-positive E. coli yields epidemiological and socioeconomic insight.** *BMC Infect Dis* 2011, **11:**238.

3. Brandal LT, Lobersli I, Stavnes TL, Wester AL, Lindstedt BA: **First report of the Shiga toxin 1 gene in sorbitol-fermenting Escherichia coli O157:H(-).** *J Clin Microbiol* 2012, **50:**1825-1826.

4. Nygård K VL, Heier BT, Bruun T, Kapperud G. **Annual report: Foodborne infections and outbreaks in 2009. Reporting system for infectious diseases (MSIS) and web-based system for outbreak warning (Vesuv)**. 2010 [In Norwegian], [<http://www.fhi.no/eway/default.aspx?pid=239&trg=Content_6466&Main_6157=6263:0:25,6493&MainContent_6263=6466:0:25,6494&Content_6466=6259:84108::0:6184:2:::0:0>].

5. MacDonald ED, P.K.; Aavitsland, P.; Brandal, L.T.; Weste,r A.L.; Vold, L.: **Implications of screening and childcare exclusion policies for children with Shiga-Toxin producing *Escherichia coli* infections: Lessons learned from an outbreak in a daycare centre, Norway, 2012.** *BMC Infectious Diseases* 2014, **Accepted.**
