# Supplementary material for: Shiga toxin-producing escherichia coli infections in Norway, 1992–2012: characterization of isolates and identification of risk factors for haemolytic uremic syndrome
Source: BMC Infect Dis. 2015 Aug 11;15:324. doi: 10.1186/s12879-015-1017-6 (PMC4531490; doi:10.1186/s12879-015-1017-6)
Supplement: Additional file 2: — STEC isolates from HUS patients included in the present study, Norway 1992–2012. Microbiological characteristics of STEC isolates (n = 26) from haemolytic uremic syndrome (HUS) patients and characteristics of patients with HUS (n = 25) in Norway from 1992–2012. [file 12879_2015_1017_MOESM2_ESM.docx]

**Additional file 2** STEC isolates from HUS patients included in the present study, Norway 1992-2012.

| **Isolate Id**^1^ | **Isolate characteristics** | | | | |  | **Patient characteristics** | | | |
| --- | --- | --- | --- | --- | --- | --- | --- | --- | --- | --- |
|  | **Year of isolation** | ***stx* status** | ***eae* status** | ***ehxA* status** | **Serotype** |  | **Season infected**^2^ | **Domestic case**^3^ | **Involved in outbreak**  **(no. of HUS cases)** |  |
| NIPH-1452/1999 | 1999 | *stx2a* | + | + | NSF O157:H7 |  | Su | ND | Yes (1) |  |
| NIPH-10212672 | 2002 | *stx1a*+*stx2a* | + | + | O111:[H8] |  | W | No | No |  |
| NIPH-10212673^4^ |  | *stx1a* | + | + | O111:[H8] |  |  |  |  |  |
| NIPH-10209818 | 2002 | *stx2a* | + | + | O26:[H11] |  | A | Yes | No |  |
| NIPH-10306923 | 2003 | *stx2a* | + | + | O103:H25 |  | A | ND | No |  |
| NIPH-11030779 | 2003 | *stx2c* | + | + | NSF O157:H7 |  | W | No | No |  |
| NIPH-11040896 | 2004 | *stx2a* | + | + | O86:H11 |  | Sp | ND | No |  |
| NIPH-11050711 | 2005 | *stx2a* | + | + | O145:H? |  | Sp | Yes | No |  |
| NIPH-11051601 | 2005 | *stx2a* | + | + | O103:H25 |  | Su | Yes | No |  |
| NIPH-11053299 | 2005 | *stx2a* | + | + | SF O157:[H7] |  | W | ND | No |  |
| NIPH-11060424 | 2006 | *stx2a* | + | + | O103:H25 |  | W | Yes | Yes (10)^5^ |  |
| NIPH-11064002 | 2006 | *stx2a* | + | + | SF O157:[H7] |  | A | Yes | No |  |
| NIPH-11072514 | 2007 | *stx2a* | + | + | O26:H11 |  | A | Yes | Yes (1) |  |
| NIPH-11072575 | 2007 | *stx2a* | + | + | O145:H? |  | A | Yes | No |  |
| NIPH-11080073 | 2008 | *stx2a* | + | + | O26:H11 |  | W | Yes | No |  |
| NIPH-11090113 | 2009 | *stx2a* | + | + | SF O157:[H7] |  | W | Yes | Yes (9) |  |
| NIPH-11090963 | 2009 | *stx2a*+*stx2c* | + | + | NSF O157:H7 |  | Sp | Yes | Yes (1) |  |
| NIPH-11091979 | 2009 | *stx2a* | + | + | O121:H19 |  | Su | ND | Yes (1) |  |
| NIPH-11092118 | 2009 | *stx2a* | + | + | O145:H? |  | Su | Yes | Yes (1) |  |
| NIPH-11101361 | 2010 | *stx2a* | + | + | O26:H11 |  | Su | ND | Yes (1) |  |
| NIPH-11101873 | 2010 | *stx2a* | + | + | O121:H? |  | A | Yes | No |  |
| NIPH-11102506^6^ | 2010 | *stx2a* | + | + | SF O157:[H7] |  | A | Yes | Yes (3) |  |
| NIPH-11110287 | 2011 | *stx2a* | + | + | NSF O157:[H7] |  | W | Yes | No |  |
| NIPH-11112203 | 2011 | *stx2a* | + | + | O145:H? |  | A | Yes | No |  |
| NIPH-11112616^6^ | 2011 | *stx2a* | + | - | SF O157:[H7] |  | A | Yes | No |  |
| NIPH-12EP000269 | 2012 | *stx2a* | + | + | O145:[H28] |  | Su | No | Yes (1) |  |

^1^Five of the isolates have been included in a previous study [[1](#_ENREF_1)]: NIPH-1020918=St. Olavs26, NIPH-11064002=St. Olavs56, NIPH-11091979=St. Olavs91, NIPH-11112203=St. Olavs164, and NIPH-11112616=St. Olavs166.

^2^Su; summer, A; autumn, W; winter, Sp; spring.

^3^ND; not determined

^4^From one of the HUS-patients two STEC isolates, with different MLVA profiles, were recovered; one carried both *stx1a* and *stx2a* while the other harboured *stx1a* only.

^5^STEC were isolated from two of 10 HUS patients, whereas the remaining eight patients yielded *stx* negative isolates.

^6^NIPH-11102506 and NIPH-11112616 showed identical MLVA-profile to the SF O157:[H7] outbreak strain from 2009 (NIPH-11090113).

**References**

1. Haugum K, Brandal LT, Lindstedt BA, Wester AL, Bergh K, Afset JE: **PCR-Based Detection and Molecular Characterization of Shiga Toxin-Producing Escherichia coli Strains in a Routine Microbiology Laboratory over 16 years.** *J Clin Microbiol* 2014, **52:**3156-3163.
