# Supplementary material for: Shiga toxin-producing escherichia coli infections in Norway, 1992–2012: characterization of isolates and identification of risk factors for haemolytic uremic syndrome
Source: BMC Infect Dis. 2015 Aug 11;15:324. doi: 10.1186/s12879-015-1017-6 (PMC4531490; doi:10.1186/s12879-015-1017-6)
Supplement: Additional file 3: — Characteristics associated with the most common STEC serogroups, Norway 1992–2012. Demographic, clinical, and virulence characteristics among STEC harbouring the most common serogroups, Norway from 1992–2012. [file 12879_2015_1017_MOESM3_ESM.docx]

**Additional file 3** Characteristics associated with the most common STEC serogroups, Norway 1992-2012.

| **Serogroup**^1^ | **No. of patients (333)** | **Age (mean)** | **Seasonal distribution/A and W**^2^ | **Domestic cases no.**^3^ | ***stx1***  **no.** | ***stx2***  **no.** | ***eae***  **no.** | ***ehxA***  **no.** | **HUS**  **no.** | **Hospitalized no.**^4^ |
| --- | --- | --- | --- | --- | --- | --- | --- | --- | --- | --- |
| NSF O157 | 103 | 33.9 | 36  p=0.02 | 32  p<0.005 | 65 | 101  p<0.005 | 103  p<0.005 | 101  p<0.005 | 4 | 57  p<0.005 |
| SFO157 | 9 | 16.6 | 8  p=0.01 | 8  p=0.02 | 1  p<0.005 | 8 | 9 | 8 | 5  p<0.005 | 7 |
| O103 | 50 | 16.3  p=0.01 | 22 | 33  p<0.005 | 47  p<0.005 | 3  p<0.005 | 49  p<0.005 | 49  p<0.005 | 3 | 19 |
| O26 | 34 | 13.5  p=0.01 | 11 | 17 | 26 | 9  p<0.005 | 34  p<0.005 | 34  p<0.005 | 4 | 12 |
| O145 | 24 | 25.0 | 13 | 15  p=0.05 | 10  p=0.03 | 14 | 23  p=0.01 | 23 | 5  p=0.03 | 15  p=0.05 |
| O91 | 13 | 25.0 | 5 | 6 | 10 | 11 | 0  p<0.005 | 11 | 0 | 3 |
| O117 | 11 | 31.6 | 6 | 3 | 10 | 1  p<0.005 | 2  p<0.005 | 2  p<0.005 | 0 | 0 |
| O121 | 7 | 2.43  p=0.02 | 3 | 6 | 1  p=0.01 | 6 | 6 | 6 | 2 | 2 |
| O113 | 6 | 40.0 | 4 | 4 | 2 | 6 | 0  p=0.01 | 4 | 0 | 3 |
| O146 | 6 | 37.5 | 4 | 3 | 6 | 4 | 0  p=0.01 | 6 | 0 | 1 |
| O111^2^ | 5 | 6.75 | 3 | 1 | 4 | 2 | 4 | 4 | 1 | 1 |

^1^Seventy (70/334, 21%) of the STEC isolates examined in this study belonged to other serogroups (including 42 non-typable STEC isolates).

^2^A; autumn, W; winter. Number of patients who fell ill during A and W, respectively.

^3^No information on travel history was available for the following patients (no.): NSFO157 (14), SFO157 (1), O103 (13), O145 (6), O26 (2), O91 (7), O117 (1), O121 (1), O113 (2), O146 (0), and O111 (0).

^4^No information on hospitalization was available from the following patients (no.): NSFO157 (9), SFO157 (0), O103 (4), O145 (2), O26 (2), O91 (2), O117 (1), O121 (1), O113 (1), O146 (0), and O111 (1).

^5^From one of the HUS patients, two O111:[H8] isolates were included, one with *stx1a*+*stx2a* and the other with *stx1a* only.
