# Supplementary material for: Shiga toxin-producing escherichia coli infections in Norway, 1992–2012: characterization of isolates and identification of risk factors for haemolytic uremic syndrome
Source: BMC Infect Dis. 2015 Aug 11;15:324. doi: 10.1186/s12879-015-1017-6 (PMC4531490; doi:10.1186/s12879-015-1017-6)
Supplement: Additional file 4 — Distribution of stx genotypes in STEC O157 compared to non-O157 STEC, Norway 1992–2012. Distribution and combination of stx1 and stx2 subtypes in STEC O157 compared to non-O157 STEC, Norway from 1992–2012. [file 12879_2015_1017_MOESM4_ESM.docx]

**Additional file 4** **Distribution of *stx* genotypes in STEC O157 compared to non-O157 STEC, Norway 1992-2012.** In general, the diversity of *stx* genotypes was higher among non-O157 STEC than in STEC O157. No statistically significant difference in the presence of *stx1* was observed between STEC O157 and non-O157, however the *stx1* subtypes *stx1c* and *stx1d* were only detected in non-O157 STEC. Nearly all O157 STEC carried *stx2*, whereas less than half of the non-O157 isolates harboured this gene. The *stx2* subtypes, *stx2c* and *stx2a*+*stx2c* were encountered only in O157 STEC. On the other hand, *stx2b* and *stx2g* were seen exclusively in non-O157 STEC group.
